# Detection of *Neisseria gonorrhoeae* and *Chlamydia trachomatis* infections in pregnant women by multiplex recombinase polymerase amplification

**DOI:** 10.1371/journal.pone.0251119

**Published:** 2021-05-04

**Authors:** Jingjing Zhai, Limin Wang, Xiaoliang Qiao, Jianping Zhao, Xuexia Wang, Xiaohong He

**Affiliations:** Department of Clinical Laboratory, Women and Infants Hospital of Zhengzhou, Zhengzhou, China; University of Texas Health Science Center at San Antonio, UNITED STATES

## Abstract

*Chlamydia trachomatis* (CT) and *Neisseria gonorrhoeae* (NG) are the main pathogenic microorganisms causing sexually transmitted infections. In this study, a multiplex thermostable recombinase polymerase amplification-lateral flow detection (RPA-LFD) assay was established, and the reaction conditions such as the ratio of primer concentration, magnesium ion concentration, amplification time and template DNA concentration in the multiplex RPA reaction were optimized. The optimized multiplex RPA-LFD method was used to detect both CT and NG positive control plasmids, and it was found that the LFD could be used to obtain visible results when the plasmid copy number was only 200. The sensitivity of the multiplex RPA-LFD method used for clinical samples was 85.62 (95% CI at 53.66–97.29) for NG detection and 90.90 (95% CI at 57.12–99.52) for CT detection.

## Introduction

*Chlamydia trachomatis* (CT) and *Neisseria gonorrhoeae* (NG) are the most common sexually transmitted pathogens and are the main contributors to sexually transmitted infections (STIs). CT infects approximately 5% to 10% of the population, with infection rates as high as 40% in patients with STIs [1]. CT and NG infections are in most cases asymptomatic and, if left untreated, can cause a variety of adverse effects and complications, such as urethritis in men or cervicitis and pelvic inflammatory syndromes in women [2] It is also one of the main causes of female infertility worldwide [3]. CT infections are particularly prevalent among young people under the age of 25 and therefore pose a major threat to the reproductive health of the population [4].

Although both of these pathogens are often widely recognized as sexually transmitted diseases, women who develop either of these sexually transmitted infections during pregnancy are at increased risk of ectopic pregnancy and can lead to adverse pregnancy outcomes such as premature rupture of membranes, preterm delivery and low birth weight of the newborn [5–7]. In addition to the impact on pregnancy outcomes, these infections can be transmitted vertically from pregnant women to their newborns during labor [8, 9], and CT and NG are also the main causes of neonatal conjunctivitis. Prevention of neonatal gonococcal ophthalmia has been practiced in the USA for over a century. Prenatal screening and treatment of these two infections in pregnant women are effective methods of preventing neonatal gonococcal ophthalmia and chlamydia infection [10]. In addition, CT can also cause neonatal pneumonia [11]. Therefore, the timely diagnosis and treatment of CT and NG for perinatal women and newborns are particularly important.

Currently, clinical diagnosis of CT and NG infections is mostly based on the PCR techniques (e.g. Abbot RealTime CT / NG assay and the Roche Cobas Amplicor CT/NG assay). Despite their high sensitivity and specificity, PCR assays require specialist handling and rely on expensive instruments such as automated sample preparation machines and real-time PCR instruments, while the time required for purification of the sample to be tested and the thermal cycling of the PCR amplification process make it impossible to reach conclusions in a short time using these methods. In recent years, the establishment of several isothermal nucleic acid amplification techniques that can be performed at constant temperature without thermal cycling has led to the rapid development of immediate diagnostic techniques for CT/GN. Recombinase polymerase amplification (RPA) is a rapidly developing nucleic acid amplification technique in recent years that does not require template denaturation and can be performed at relatively low and constant temperatures (38°C to 42°C). It relies on recombinase complexes from T4 phages to introduce primers into specific DNA sites, and amplification reactions are catalyzed by strand-substitution DNA polymerase in concert with single-stranded DNA-binding proteins (SSBs) [12]. The sensitivity of RPA is comparable to that of PCR. Some researches show that specific amplification is possible when the copy number of the template in the reaction system is as low as 10. In addition, RPA can rapidly produce detectable amplification signals within 10 minutes, even at very low template concentrations [13].

RPA amplification products can be detected by various methods, such as flocculation assay detection, lateral flow assay detection (LFD), electrochemical detection, chemiluminescent detection, and silicon microring resonator (SMR)-based photonic detection. Among these methods, the lateral flow immunoassay is widely used because of its rapid, sensitive, specific, and cost-effective nature. In this assay, the amplification product is labelled in the amplification reaction via the forward and reverse primers by two different antigen molecules, one of which (usually digoxin) binds to a specific antibody coupled to the colloidal gold nanoparticles, and the other to a specific antibody immobilized on the surface of the lateral flow strip detection line, resulting in a colored signal on the detection line that can be determined by visual observation [14]. In this study, we optimized the reaction conditions for RPA and developed a multiplex thermostatic RPA-LFD assay, which was successfully used to detect CT and NG in clinical samples.

## Materials and methods

### Ethics approval and consent to participate

The procedures in the present study were approved by the Ethics Committee of Women and Infants Hospital of Zhengzhou (Zhengzhou, China). All research complied with the declaration of Helsinki and the relevant rules of the Ethics Committee. All patients (or their parent or legal guardian) provided written informed consent for their data to be used in the study.

### Preparation of positive control plasmids

The *N*. *gonorrhoeae* strain (BNCC337543) and *C*. *trachomatis* (ATCC^®^ VR-348BD) strain used in this study were purchased from the BeNa Strain Preservation Biotechnology Research Center (Beijing, China). In this experiment, the *CDS2* gene in *C*. *trachomatis* and *FIT* gene in *N*. *gonorrhoeae* were designated as characteristic genes. The pGL3-CT and pGL3-NG positive control plasmids were constructed by ligating the clones of these two genes between XhoI restriction endonuclease sites located downstream of the promoter on the pGL3 vector (Promega, Madison, WI) and were validated by PCR and restriction digestion analysis. (The primer information is shown in Table 1).

**Table 1:** Names and sequences of primers used in this study.

Species	Primers	Sequences	Modification
***N*. *gonorrhoeae***	PCR-FP	5’-TTACTCGAGCCGCGTACGTCTTCCAGCT -3’	-
	PCR-RP	5’-TTACTCGAGCAACGCAATCAAATTCCGTGCGC -3’	-
	RPA-FP	5’-CAACGCAATCAAATTCCGTGCGCGAGCCGCAG-3’	Biotin
	RPA-RP	5’-CCGCGTACGTCTTCCAGCTCAACACCTCCGAT-3’	Digoxin
***C*. *trachomatis***	PCR-FP	5’-GTCCTCGAGATCCTCAGAAGTTTATGC -3’	-
	PCR-RP	5’-GTCCTCGAGCTGTGACCTTCATTATGT -3’	-
	RPA-FP	5’-TTAAGGCAAATCGCCCGCACGTTCTCTCAAGC-3’	FAM
	RPA-RP	5’-CAAATATCATCTTTGCGGTTGCGTGTCCTGTG-3’	Digoxin

### RPA primer design and construction

The highly conserved sequence regions of *N*. *gonorrhoeae FIT* and *C*. *trachomatis CDS2* genes were sought by using the Basic Local Alignment Search Tool (BLAST; http://blast.ncbi.nlm.nih.gov). The conservation of the *CDS2* amplicon among 21 different serovars is shown in S1 Raw images.

The primers were designed based on the conserved regions in the CDS2 gene of *C*. *trachomatis* and the FIT gene of *N*. *gonorrhoeae* in accordance with the requirements of the TwistAmp^®^ COMBINED INSTRUCTION MANUAL, and the candidate primers were selected according to the following principles: primer length of 30–35 bases, amplification product size of 100–200 base pairs, absence of guanine repeats in the 3–5 bases at the 5’ end of the primer, and the last 3 bases at the 3’ end being predominantly guanine and cytosine. OligoAnalyser was further used to analyze the above candidate primers sequences to screen out those who might form dimers, hairpin structures or other secondary structures. (http://www.idtdna.com/analyzer/Applications/OligoAnalyzer). The primer sequences that passed the screening were used for primer construction, during which the forward primer for the CDS2 gene was labelled at the 5’ with Biotin, the forward primer for the Fit gene was labelled at the 5’ with FAM, and the two reverse primers were labelled at the 5’ with Digoxin. Sequence of NG-RPA primer was from Kersting’ article [15]. The primers were synthesized by Sangon Biotech (Shanghai, China) Co., Ltd., and the primer information is presented in Table 1.

### Establishment and optimization of a multiplex RPA reaction system

The reaction system (50 μL) was established according to the TwistAmp Basic kit (TwistDX, UK) instructions as follows: 2.1 μL forward primer mix; 2.1 μL reverse primer mix, 25 μL of 2 × Reaction buffer (TwistDX), 1 μL template DNA (CT and NG), enzyme, finalized to 47.5 μL with double-distilled water and 2.5 μL of magnesium acetate before the start of the reaction. The mixture was incubated at 38 °C for RPA with gentle agitation every 4 minutes and then incubated at 50 °C for 10 min to terminate the reaction. The multiplex RPA reaction system was optimized in terms of: magnesium acetate concentration (2.8, 5.6, 11.2, 16.8, 22.4 or 28 mM), primer concentration (for NG and CT at 500 nM + 100 nM, 400 nM + 200 nM, 300 nM + 300 nM, 200 nM + 400 nM or 100 nM + 500 nM) and reaction times (2.5, 5, 10, 15 or 20 min).

### Lateral flow assay detection (LFD)

The RPA amplification reaction results in a double-stranded DNA product with antigen markers corresponding to the primers on the sense and antisense strands, which first bind to the murine anti-digoxin monoclonal antibodies coupled to the colloidal gold particles on the coupling pad and then bind to the anti-biotin antibody or anti-FAM antibody immobilized on two different detection lines, resulting in the color development of the corresponding detection line. An anti-mouse IgG antibody is immobilized on the quality control line above the two detection lines, and excess antibody-colloidal gold particles bind to it as the sample flows through, leading to the color development on the quality control line.

### Sensitivity experiments of the combined multiplex RPA-LFD detection system

To evaluate the sensitivity of the multiplex RPA-LFD assay system constructed in this study, the CT and NG positive control plasmids described above were diluted with TE-buffer to the concentration of 25, 50, 100, 200 and 400 copies/μL, and 1 μL of the positive control plasmids was subject to the multiplex RPA-LFD assay to analyze the lowest template concentration that could be detected by this method.

### Multiple RPA-LFD test analysis on clinical samples

In this study, the first-void urine samples were collected from 79 pregnant women (22–35 years old) in Sep and Oct of 2019. Participants collected their own first segment urine samples in the morning under the direction of their clinician and stored them in clean polypropylene containers without preservatives, with an average sample volume of 20 ml. Samples were temporarily stored for less than 1 day at 4°C after collection and heated at 90°C for 5 min before DNA extraction, and total DNA was extracted for RPA using the QIAamp DNA Mini Kit (Qiagen, Germany); the amplification products were subsequently tested using the LFD system. This part of the study used a fluorescent PCR method-based CT/NG/UU nucleic acid detection kit as a control method; the Path-N. gonorrhoeae-standard and Path-C. trachomatis-standard (Primer-design Co., Ltd, Camberley, UK) were used to establish positive control sample, and sterile saline was used as a negative control sample.

### Data analysis

The VassarStats calculator (http://www.vassarstats.net/clin1.html) was used to calculate the specificity, sensitivity and 95% confidence interval.

## Results

### Primer specificity check and positive standard testing

**Fig 1:**
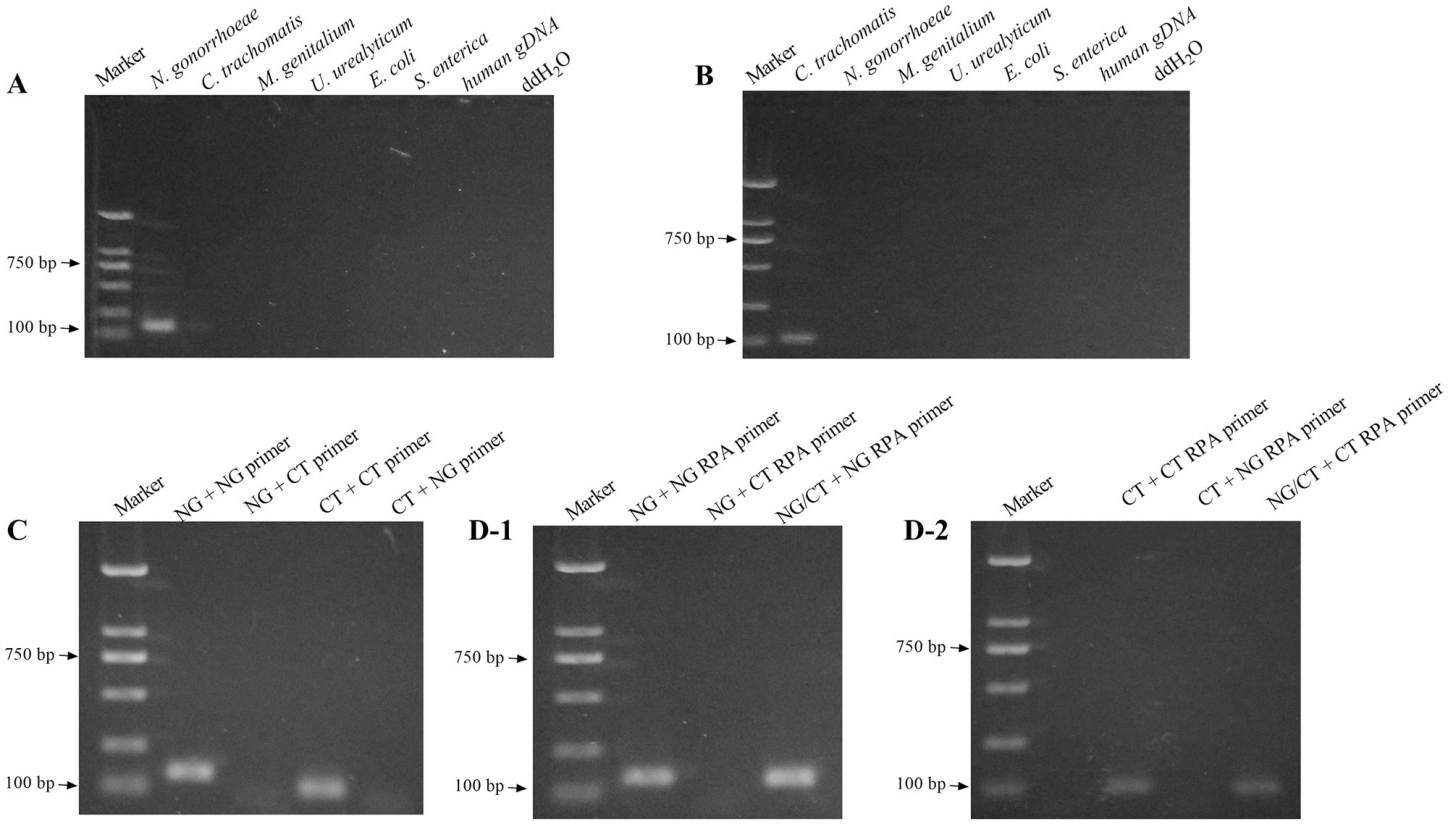
Primer specificity assay and positive control plasmid validation. A and B: RPA was performed using NG-RPA primer pairs and CT-RPA primer pairs with six common pathogenic bacteria including NG and CT and human genomic DNA as templates, and the amplification results were examined by agarose gel electrophoresis. C: NG and CT genomic DNA was subject to PCR using NG-PCR primer pairs or CT-PCR primer pairs, and the amplification results were examined by agarose gel electrophoresis. The primer pairs and templates are grouped as indicated in the figure. D-1 and D-2: NG and CT genomic DNA were used as templates for RPA using NG-RPA primer pairs or CT-RPA primer pairs, and the amplification results were examined by agarose gel electrophoresis. The primer pairs and templates were grouped as indicated in the figure.

In this study, we used CT, NG and four other common bacterial and human genomic DNA as templates for RPA and tested the amplification products by agarose gel electrophoresis to evaluate the specificity of the primers. The results showed that the genomic DNA of CT or NG strains could be amplified by RPA with the corresponding primers used in this study, whereas the amplified products could not be obtained by the same experimental method using the other four strains or human genomic DNA as templates, as shown in Fig 1A and 1B, suggesting a high specificity of the primers used in this study. On this basis, we performed PCR (Fig 1C) and RPA (Fig 1D) using the CT and NG positive control plasmids as templates, respectively, and detected the amplified products by agarose gel electrophoresis. The results showed that the amplification products could be obtained by PCR and RPA respectively using primers corresponding to the positive control plasmids, while the amplification products could not be obtained when the primers did not correspond to the positive control plasmids, suggesting that the positive control plasmids were successfully constructed.

### Optimization of multiple RPA reaction system

**Fig 2:**
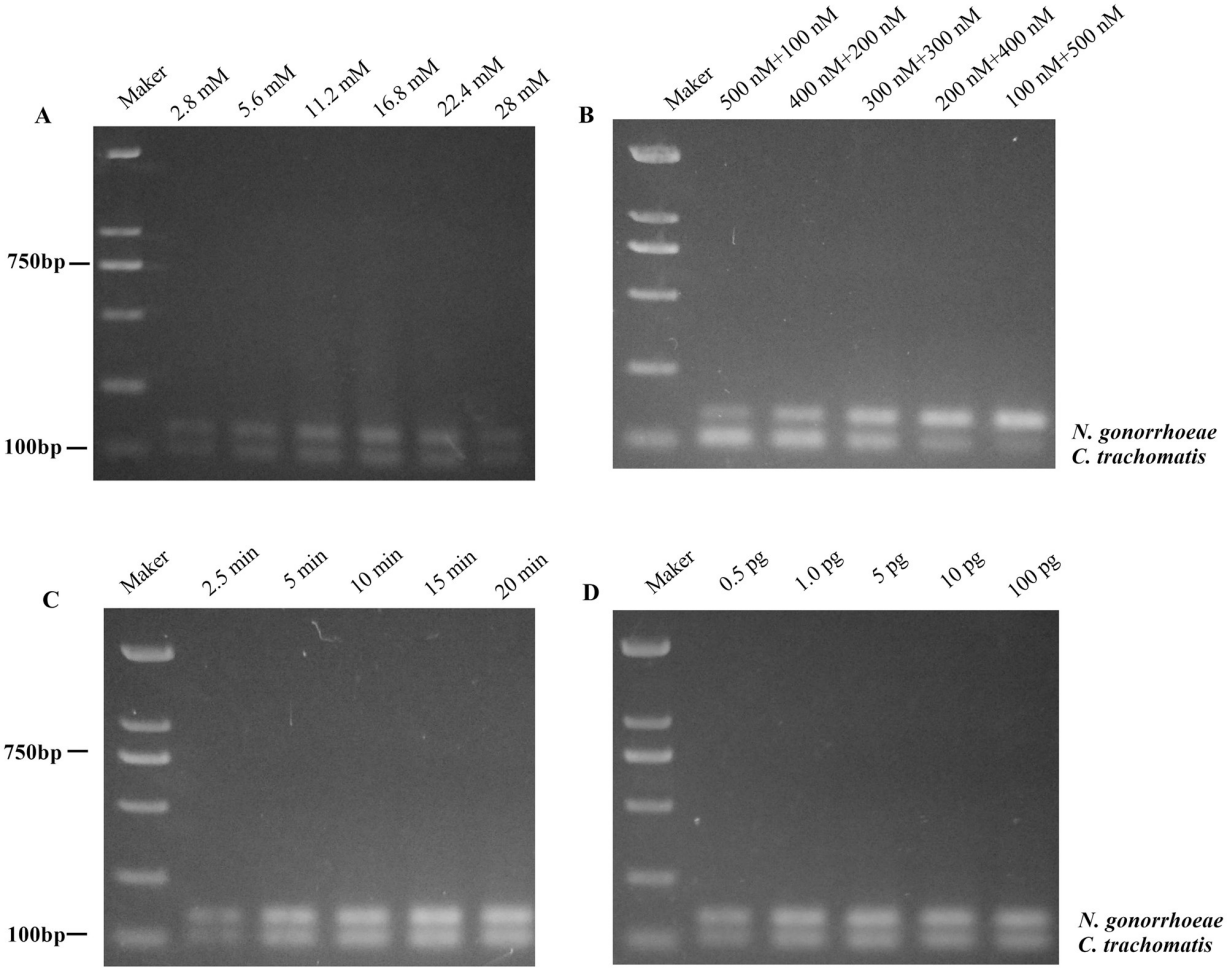
Optimization of multiple RPA reaction system. A: Comparison of amplification efficiency of multiplex RPA at different magnesium ion concentrations (38°C, 20 min, CT 200 nM + NG 200 nM); B: Comparison of amplification efficiency of multiplex RPA at different combinations of primer pair concentrations (38°C, 20 min, 16.8 nM Mg^2+^); C: Comparison of amplification yields of multiplex RPA at different amplification times (38°C, 16.8 nM Mg^2+^, CT 300 nM + NG 300 nM); D: Comparison of amplification efficiency of multiplex RPA at different template concentration conditions (38°C, 10 min, 16.8 nM Mg^2+^, CT 300 nM + NG 300 nM).

In this study we analyzed the effects of magnesium ion concentration, primer concentration, reaction time and the concentration of template DNA on amplification efficiency during PRA. We first compared the amplification efficiencies at different magnesium ion concentrations (2.8, 5.6, 11.2, 16.8, 22.4 and 28 mM) by agarose gel electrophoresis. The PRA was performed at 38 °C for 20 min using 1 pg CT/NG gDNA as the template and 200 nM of CT-RPA primer pairs plus 200 nM of NG-PRA primer pairs. The results showed that the amplification efficiency increased with increasing Mg ion concentration at low levels; when the Mg ion concentration was higher than 16.8 mM, the amplification efficiency decreased with further increase in Mg ion concentration, as shown in Fig 2A. We therefore considered 16.8 mM as the optimal concentration of magnesium ion.

Since different target genes have different amplification efficiencies, when primer concentrations are high, different primers may affect each other’s amplification efficiency; in order to achieve similar amplification efficiencies, we set up five different sets of primer concentration combinations (500 nM+100 nM, 400 nM+200 nM, 300 nM+300 nM, 200 nM+400 nM, 100 nM+500 nM) for RPA to determine the optimal primer concentrations. RPA was performed at 38°C for 20 minutes with the magnesium ion concentration at 16.8 mM using 1 pg of CT/NG gDNA as the template, and the amplification products were examined by agarose gel electrophoresis. The results showed that the amplification efficiency of CT and NG was more balanced and the bands were obvious when the primer concentrations were 300 nM for CT-RPA primers + 300 nM for NG-RPA primers or 400 nM for CT-RPA primers + 200 nM for NG-RPA primers, as shown in Fig 2B.

On this basis, we further investigated the effect of different amplification times (2.5, 5, 10, 15 or 20 min) on the amount of amplified product. RPA was performed at 38°C with the magnesium ion concentration at 16.8 mM using 1 pg of CT/NG gDNA as the template and 400 nM of CT-RPA primers plus 200 nM of NG-RPA primers, and the amplification products were examined by agarose gel electrophoresis. The results showed that the specific amplification products could be detected by agarose gel electrophoresis after 5 min of RPA; the brightness of the bands increased with longer reaction time, indicating that longer reaction time could yield more amplification products, as shown in Fig 2C. However, too long reaction time was not suitable for application in rapid detection, we therefore decided to set the reaction time for RPA at 10 min.

Based on the reaction conditions determined above, we performed the RPA reactions at different template concentrations using CT and NG genomic DNA as templates. The results showed that amplification bands could already be observed when the template DNA content was only 0.5 pg, and the bands were clear and obvious when the template DNA content was 1 pg, as shown in Fig 2D.

### Establishment and optimization of LFD detection system

**Fig 3:**
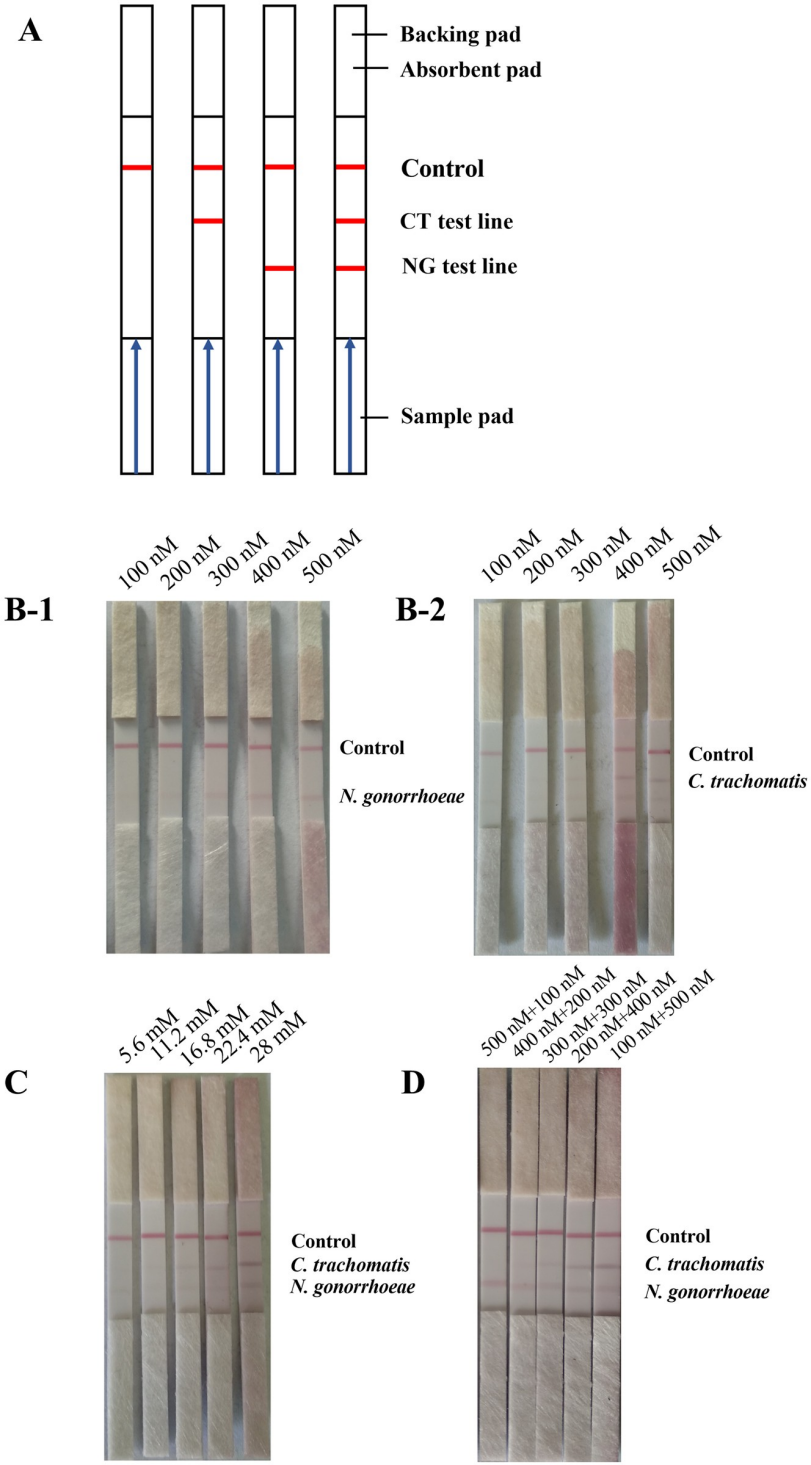
Construction and optimization of LFD detection system. A: Schematic diagram of the LFD system with blue arrows indicating the direction of liquid flow and short red lines marking the positions of the detection and quality control lines; B: The influence of difference in RPA primer concentrations on the assay results of the multiplex of LFD-RPA method (38°C, 10 min, 16.8 nM Mg^2+^), B-1: NG, B-2: CT; C: Effect of different magnesium ion concentrations on the assay results of the multiplex RPA-LFD method (38°C, 10 min, CT 400 nM + NG 200 nM); D: Effect of different primer concentration combinations on the assay results of the multiplex RPA-LFD method (38°C, 10 min, 16.8 nM Mg^2+^).

As shown in Fig 3A, the LFD system is mainly composed of a sample pad, a conjugate pad, an absorbent pad, a backing pad, and a nitrocellulose membrane with two detection lines and one control line. We used this LFD system to examine the amplification products obtained by RPA under different experimental conditions. The results showed that, when RPA was performed with one pair of primers, the detection of their respective amplification products in the LFD system could be clearly observed by the naked eye when the NG-RPA primer concentration reached 300 nM or when the CT-RPA primer reached 400 nM, as shown in Fig 3B. When RPA was performed at this primer concentration, the products amplified from the genomic DNA of both pathogens were clearly visible to the naked eye when the magnesium ion concentration reached 16.8 mM, as shown in Fig 3C. We further compared the LFD results of the amplification products by RPA using different primer concentration ratios, and the results showed that, when the concentration of CT-RPA primer pair was 400 nM and the concentration of NG-RPA primer pair was 200 nM, the products amplified with the genomic DNA of both pathogens as templates could be clearly observed by the naked eye in the detection results of LFD, as shown in Fig 3D. Based on these results, we set the multiplex RPA reaction conditions for LFD detection to a magnesium ion concentration of 16.8 mM, CT-RPA primer pair concentration of 400 nM, NG-RPA primer pair concentration of 200 nM, and amplification time of 10 min.

### Sensitivity evaluation of multiple LFD-RPA assay

**Fig 4:**
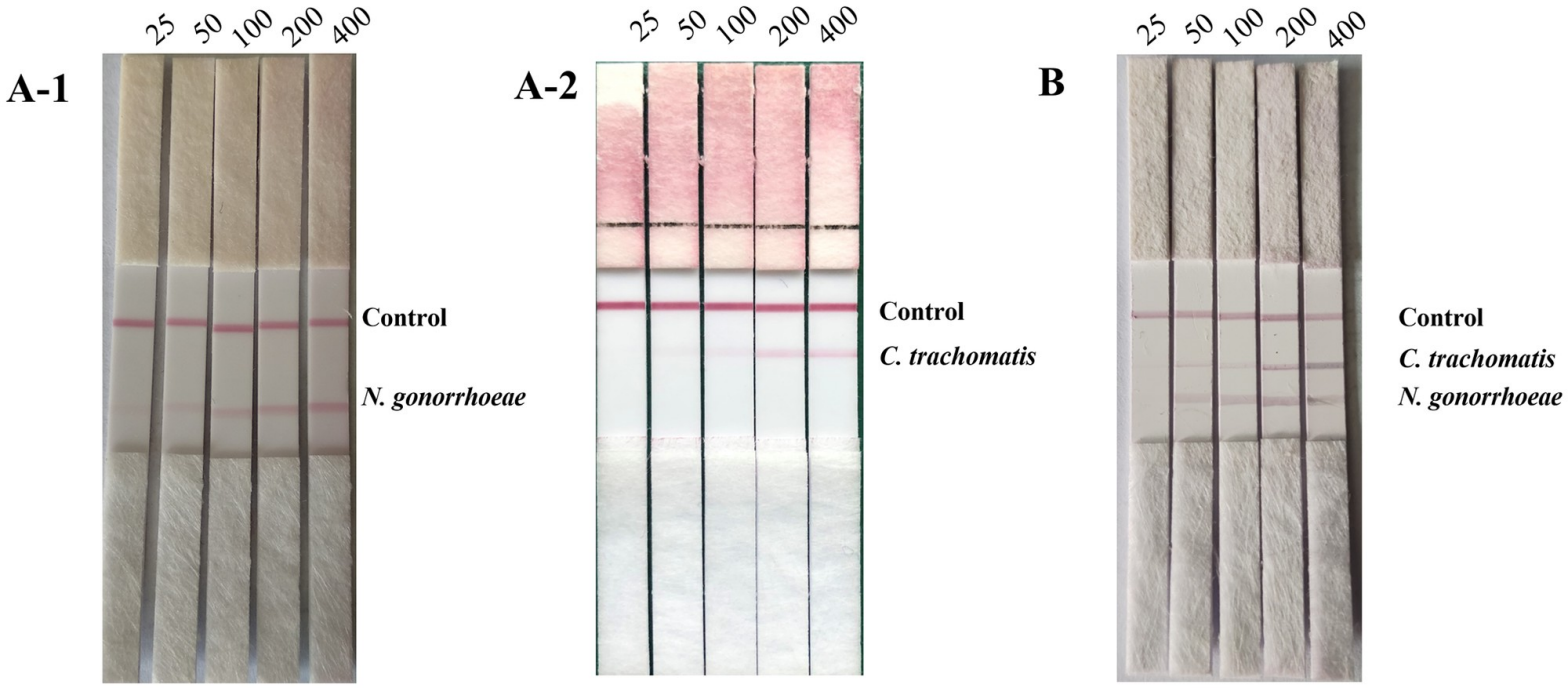
Sensitivity test of multiple LFD-RPA detection. A: Assay results when NG or CT positive control plasmids of different copy numbers were used as templates, respectively; B: Assay results when NG or CT positive control plasmids of different copy numbers were used in the same system. The RPA was performed at 38°C for 10 min with magnesium ion concentration at 16.8 nM, CT-RPA primer concentration at 400 nM and NG-RPA primer concentration at 200 nM.

We used the multiplex RPA-LFD assay established in this study to detect CT and NG positive control plasmids with different copy numbers in order to evaluate the sensitivity of this method. We found that the results of the multiplex RPA-LFD assay were clearly visible to the naked eye when the copy number of the NG positive control plasmid was equal to or higher than 50, and the same results were obtained when the copy number of the CT positive control plasmid was equal to or higher than 200, as shown in Fig 4A. We then performed multiplexed RPA-LFD assays on both NG and CT positive control plasmids and found that the results were clearly visible to the naked eye by the multiplexed RPA-LFD method when the copy number of both NG and CT positive control plasmids was equal to or higher than 200, as shown in Fig 4B.

### Detection of NG and CT in clinical samples using multiplex RPA-LFD method

**Fig 5:**
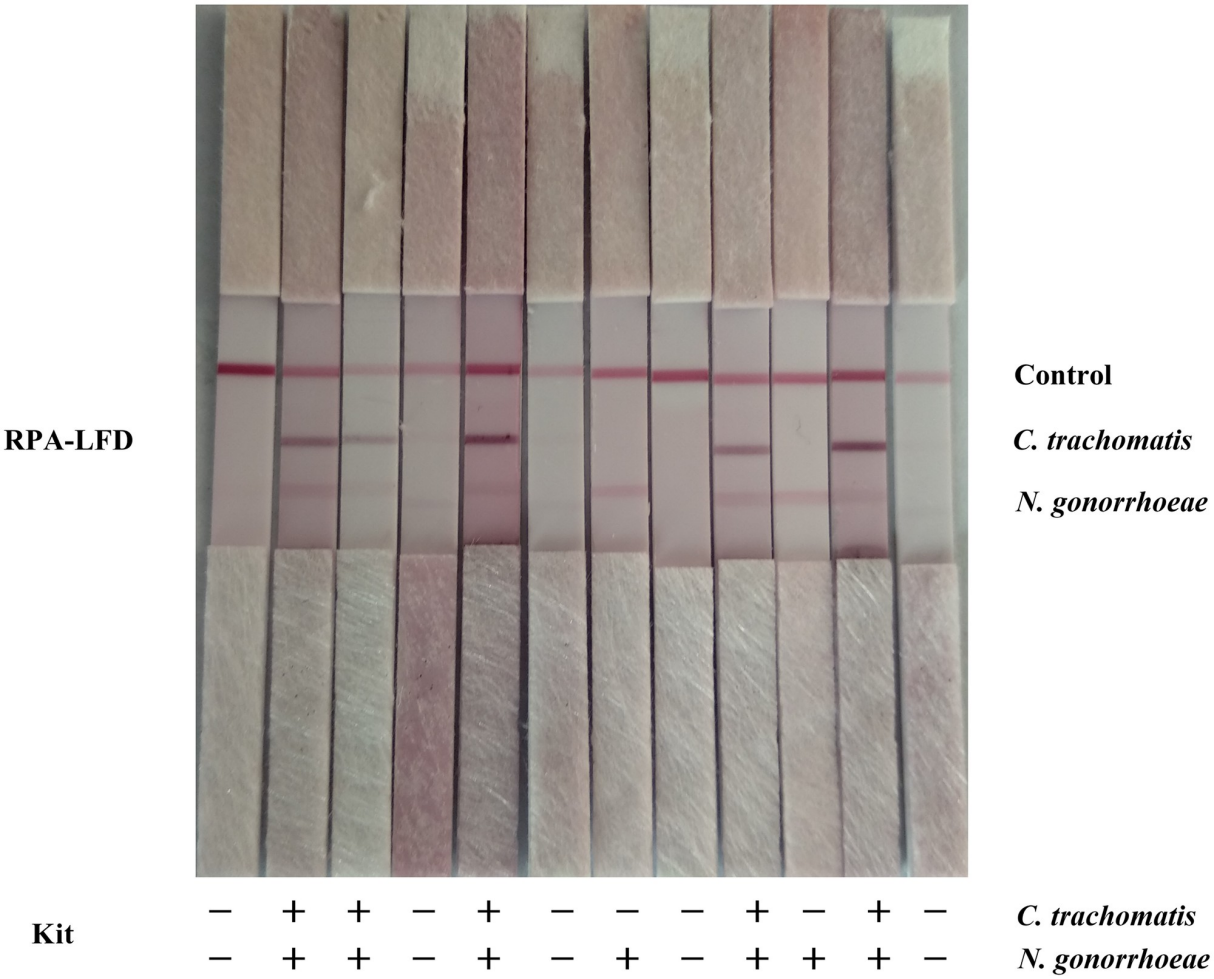
Randomly picked results of the multiplex RPA-LFD assay when used to assay clinical samples.

To evaluate the sensitivity of the multiplex LFD-RPA assay when used for the detection of clinical samples, the urine samples from 79 subjects were collected and tested using the commercially purchased CT/NG detection kits and by the multiplex LFD-RPA assay established in this study in parallel. We found that, in the 13 NG-positive samples detected by the commercially purchased CT/NG detection kit, 11 of which were detected by the multiplex RPA-LFD method while 2 were not; the sensitivity of the multiplex RPA-LFD assay for detecting NG in clinical samples was 84.62 (95% CI, 53.66–97.29) compared with that of the commercially purchased CT/NG detection kit. Of the 11 CT-positive samples detected by the commercially purchased CT/NG detection kit, 10 were detected by the multiplex RPA-LFD method while 1 was not, with a sensitivity of 90.90 (95% CI, 57.12–99.52). Among the 6 CT/NG co-positive samples detected by the commercially purchased kit, 5 were detected by the multiplex RPA-LFD method while 1 was not, with a sensitivity of 83.33 (95% CI, 36.48–99.12), as shown in Table 2 and Fig 5.

**Table 2:** Sensitivity and specificity of multiple RPA-LFD assay.

Sample	Results	Detection kit		Sensitivity (%)	Specificity (%)
		Positive	Negative		
NG	Positive	11	0	84.62 (95% CI, 53.66–97.29)	100 (95% CI, 93.15–100)
	Negative	2	66		
CT	Positive	10	0	90.90 (95% CI, 57.12–99.52)	100 (95% CI, 93.33–100)
	Negative	1	68		
NG+CT	Positive	5	0	83.33 (95% CI, 36.48–99.12)	100 (95% CI, 93.77–100)
	Negative	1	73		

## Discussion

The risk of blindness caused by CT/NG conjunctivitis depends on the local medical conditions, especially in developing countries and or in some areas with limited access to medical care, vertical transmission from perinatal women can cause neonatal blindness in up to 3% of children with CT/NG infectious conjunctivitis [16]. A study on prenatal screening for CT/NG showed that there was no significant association between the lack of self-reported symptoms of sexually transmitted infections (such as abnormal vaginal discharge, lower abdominal pain and/or painful urination) and a positive diagnosis of CT/NG in subjects, and that a large proportion of patients with CT/NG infection were asymptomatic; therefore, the CT/NG infection in pregnant women may not be adequately identified by general symptoms of sexually transmitted infections [17]. In addition, the CT/NG infection may also lead to other inflammatory diseases of the female reproductive system such as cervicitis or pelvic inflammation as well as female infertility [2, 3]. Hence, the CT/NG testing is particularly important for women, especially the perinatal women.

Current clinical use of CT/NG diagnostic techniques based on fluorescent PCR methods, while allowing for highly specific pathogen detection, are often costly and do not allow for immediate diagnosis after infection due to insufficient sensitivity. For developing countries or other medically disadvantaged areas, there is an urgent need for a low-cost and rapid CT/NG diagnostic technique that can be performed at the point of care (POC). The World Health Organization (WHO) provides guidelines for the development of POC for the detection of STI, a major health problem for diseases such as Chlamydia and HIV in developing countries as well as in developed countries [18]. These rules are called ASSURED: Affordable, for those at risk of infection; Sensitive, minimal false negatives; Specific, minimal false positives; User-friendly, minimal steps to carry out test; Rapid & Robust, short turnaround time and no need for asking storage; Equipment-free, no complex equipment; Delivered, to end users [19]. Obviously, it is difficult to meet all the technical requirements at this stage, but some research investigations show that the factors that patient value more are low cost, high sensitivity and specificity, and short waiting time.

The RPA-FLD system performed this experiment is suitable for kit-based POC detection. Its operation is simple, no high demands to operating personnel and equipment, strip of low cost, and high sensitivity, high specificity, at the same time, patients in clinic for the first time can get the test results, without having to pay a return visit. It could potentially limit onward transmission and reduce rates of sequelae.

Katrin Krolov *et al*. [14] have developed a CT testing system based on the RPA-LFD, its sensitivity to clinical samples of 83% (95% CI, 51%-97%), specificity of 100% (95% CI, 92%-100%). This system is also based on the RPA amplification of CDS2 conservative sequence. Compared with the previous detection system, our experiment combined two kinds of the STI pathogens, CT and NG, detection into one process, whether the RPA process or LFD process, it undoubtedly improves the detection efficiency. However, in multiple reaction systems, different templates and amplified primers may influence each other, or for this reason, the minimum template amount (200 copies) that can be detected in the simultaneous detection of two pathogens in this experiment is higher than that for a single pathogen (50 copies).

Factors such as primer design, magnesium ion content, crowding agent and reaction time may all affect the outcome of RPA. The RPA amplification relies on a viscous crowding agent for optimal nucleic acid amplification, which prevents spontaneous recombinase-primer dissociation with the presence of the SSBs required for amplification. However, the reaction temperature of RPA is commonly low (38–42°C), and the mixing by thermal convection in the reaction system is not strong, local depletion of the reagents in the more reactive regions of the system might therefore block the RPA from continuing. Manual mixing after every 3–6 minutes of incubation can re-homogenize the reaction system and improve amplification efficiency [20]. The time required to amplify the template to a detectable level depends on the amount of templates, but a reaction time of 20 minutes is usually sufficient, and some studies have shown that the amplification time required to obtain a detectable amplification productcan be as low as 3–4 minutes [21]. Excessive incubation time are generally considered detrimental to RPA reactions; for the liquid-phase RPA, the recombinase can consume all available ATP in the reaction system within 25 minutes [22]. It has been shown that the amplified product of the RPA reaction can be detected by LFD method with a sensitivity of 83% and a specificity of 100% using only heat-treated urine samples as templates, which undoubtedly simplifies the operational steps of the assay and significantly reduces the overall detection time, facilitating the establishment and development of CT/NG POC diagnosis in areas where medical supplies are scarce [14]. In this study, we have successfully established a multiplex RPA-LFD method and optimized the reaction conditions for multiplex RPA. Using this method, positive amplification products of both CT and NG could be successfully detected at a template copy number of only 200, and CT/NG infection was successfully detected in clinical samples with high specificity and sensitivity. The results of this study will contribute to the development and popularization of CT/NG clinical diagnosis.

## Supporting information

S1 Raw images (ZIP)

## References

[1] Mylonas I. Female genital Chlamydia trachomatis infection: where are we heading? Arch Gynecol Obstet 2012;285(5):1271–85. 10.1007/s00404-012-2240-7 22350326

[2] Kalwij S, Macintosh M, Baraitser P. Screening and treatment of Chlamydia trachomatis infections. BMJ 2010;340:c1915. 10.1136/bmj.c1915 20410164

[3] Baud D, Regan L, Greub G. Emerging role of Chlamydia and Chlamydia-like organisms in adverse pregnancy outcomes. Curr Opin Infect Dis 2008;21(1):70–6. 10.1097/QCO.0b013e3282f3e6a5 18192789

[4] Ginocchio CC, Chapin K, Smith JS, Aslanzadeh J, Snook J, Hill CS, et al. Prevalence of Trichomonas vaginalis and coinfection with Chlamydia trachomatis and Neisseria gonorrhoeae in the United States as determined by the Aptima Trichomonas vaginalis nucleic acid amplification assay. J Clin Microbiol 2012;50(8):2601–8. 10.1128/JCM.00748-12 22622447 PMC3421522

[5] Mullick S, Watson-Jones D, Beksinska M, Mabey D. Sexually transmitted infections in pregnancy: prevalence, impact on pregnancy outcomes, and approach to treatment in developing countries. Sex Transm Infect 2005;81(4):294–302. 10.1136/sti.2002.004077 16061534 PMC1745010

[6] Johnson HL, Ghanem KG, Zenilman JM, Erbelding EJ. Sexually transmitted infections and adverse pregnancy outcomes among women attending inner city public sexually transmitted diseases clinics. Sex Transm Dis 2011;38(3):167–71. 10.1097/OLQ.0b013e3181f2e85f 20852454

[7] Chow JM, Kang MS, Samuel MC, Bolan G. Assessment of the Association of Chlamydia trachomatis Infection and Adverse Perinatal Outcomes with the Use of Population-Based Chlamydia Case Report Registries and Birth Records. Public Health Rep 2009;124 Suppl 2:24–30. 10.1177/00333549091240S205 27382651 PMC2775397

[8] Honkila M, Wikstrom E, Renko M, Surcel HM, Pokka T, Ikaheimo I, et al. Probability of vertical transmission of Chlamydia trachomatis estimated from national registry data. Sex Transm Infect 2017;93(6):416–420. 10.1136/sextrans-2016-052884 28228485

[9] Chang JH, Huang YL, Chen CC, Li SY. Vertical transmission of Neisseria gonorrhoeae to a female premature neonate with congenital pneumonia. J Formos Med Assoc 2013;112(10):648–9. 10.1016/j.jfma.2013.04.008 23727313

[10] Hammerschlag MR. Chlamydial and gonococcal infections in infants and children. Clin Infect Dis 2011;53 Suppl 3:S99–102. 10.1093/cid/cir699 22080275

[11] Beem MO, Saxon EM. Respiratory-tract colonization and a distinctive pneumonia syndrome in infants infected with Chlamydia trachomatis. N Engl J Med 1977;296(6):306–10. 10.1056/NEJM197702102960604 831128

[12] Piepenburg O, Williams CH, Stemple DL, Armes NA. DNA detection using recombination proteins. PLoS Biol 2006;4(7):e204. 10.1371/journal.pbio.0040204 16756388 PMC1475771

[13] Euler M, Wang Y, Otto P, Tomaso H, Escudero R, Anda P, et al. Recombinase polymerase amplification assay for rapid detection of Francisella tularensis. J Clin Microbiol 2012;50(7):2234–8. 10.1128/JCM.06504-11 22518861 PMC3405570

[14] Krolov K, Frolova J, Tudoran O, Suhorutsenko J, Lehto T, Sibul H, et al. Sensitive and rapid detection of Chlamydia trachomatis by recombinase polymerase amplification directly from urine samples. J Mol Diagn 2014;16(1):127–35. 10.1016/j.jmoldx.2013.08.003 24331366

[15] Kersting S, Rausch V, Bier FF, von Nickisch-Rosenegk M. Multiplex isothermal solid-phase recombinase polymerase amplification for the specific and fast DNA-based detection of three bacterial pathogens. Mikrochim Acta 2014;181(13–14):1715–1723. 10.1007/s00604-014-1198-5 25253912 PMC4167443

[16] Schaller UC, Klauss V. Is Crede’s prophylaxis for ophthalmia neonatorum still valid? Bull World Health Organ 2001;79(3):262–3. 11285676 PMC2566367

[17] Wynn A, Ramogola-Masire D, Gaolebale P, Moshashane N, Sickboy O, Duque S, et al. Prevalence and treatment outcomes of routine Chlamydia trachomatis, Neisseria gonorrhoeae and Trichomonas vaginalis testing during antenatal care, Gaborone, Botswana. Sex Transm Infect 2018;94(3):230–235. 10.1136/sextrans-2017-053134 29097418 PMC6117829

[18] Peeling RW, Holmes KK, Mabey D, Ronald A. Rapid tests for sexually transmitted infections (STIs): the way forward. Sex Transm Infect 2006;82 Suppl 5:v1–6. 10.1136/sti.2006.024265 17151023 PMC2563912

[19] St JA, Price CP. Existing and Emerging Technologies for Point-of-Care Testing. Clin Biochem Rev 2014;35(3):155–67. 25336761 PMC4204237

[20] Lillis L, Siverson J, Lee A, Cantera J, Parker M, Piepenburg O, et al. Factors influencing Recombinase polymerase amplification (RPA) assay outcomes at point of care. Mol Cell Probes 2016;30(2):74–8. 10.1016/j.mcp.2016.01.009 26854117 PMC4818709

[21] Xia X, Yu Y, Weidmann M, Pan Y, Yan S, Wang Y. Rapid detection of shrimp white spot syndrome virus by real time, isothermal recombinase polymerase amplification assay. PLoS One 2014;9(8):e104667. 10.1371/journal.pone.0104667 25121957 PMC4133268

[22] Lobato IM, O’Sullivan CK. Recombinase polymerase amplification: Basics, applications and recent advances. Trends Analyt Chem 2018;98:19–35. 10.1016/j.trac.2017.10.015 32287544 PMC7112910

