# Correction: Detection of *Neisseria gonorrhoeae* and *Chlamydia trachomatis *infections in pregnant women by multiplex recombinase polymerase amplification

**DOI:** 10.1371/journal.pone.0271836

**Published:** 2022-07-15

**Authors:** Jingjing Zhai, Limin Wang, Xiaoliang Qiao, Jianping Zhao, Xuexia Wang, Xiaohong He

The following information is missing from the Funding statement: The authors received funding from the Science and Technology Development Plan Project of Henan Province No.182102310481.

In the last sentence of the Abstract, the number 85.62 is incorrect. It should be 84.62. The correct sentence is: The sensitivity of the multiplex RPA-LFD method used for clinical samples was 84.62 (95% CI at 53.66–97.29) for NG detection and 90.90 (95% at 57.12–99.52) for CT detection.
