# Supplementary figures and images for: Detection of Neisseria gonorrhoeae and Chlamydia trachomatis infections in pregnant women by multiplex recombinase polymerase amplification
Source: PLoS One. 2021 May 4;16(5):e0251119. doi: 10.1371/journal.pone.0251119 (PMC8096098; doi:10.1371/journal.pone.0251119)

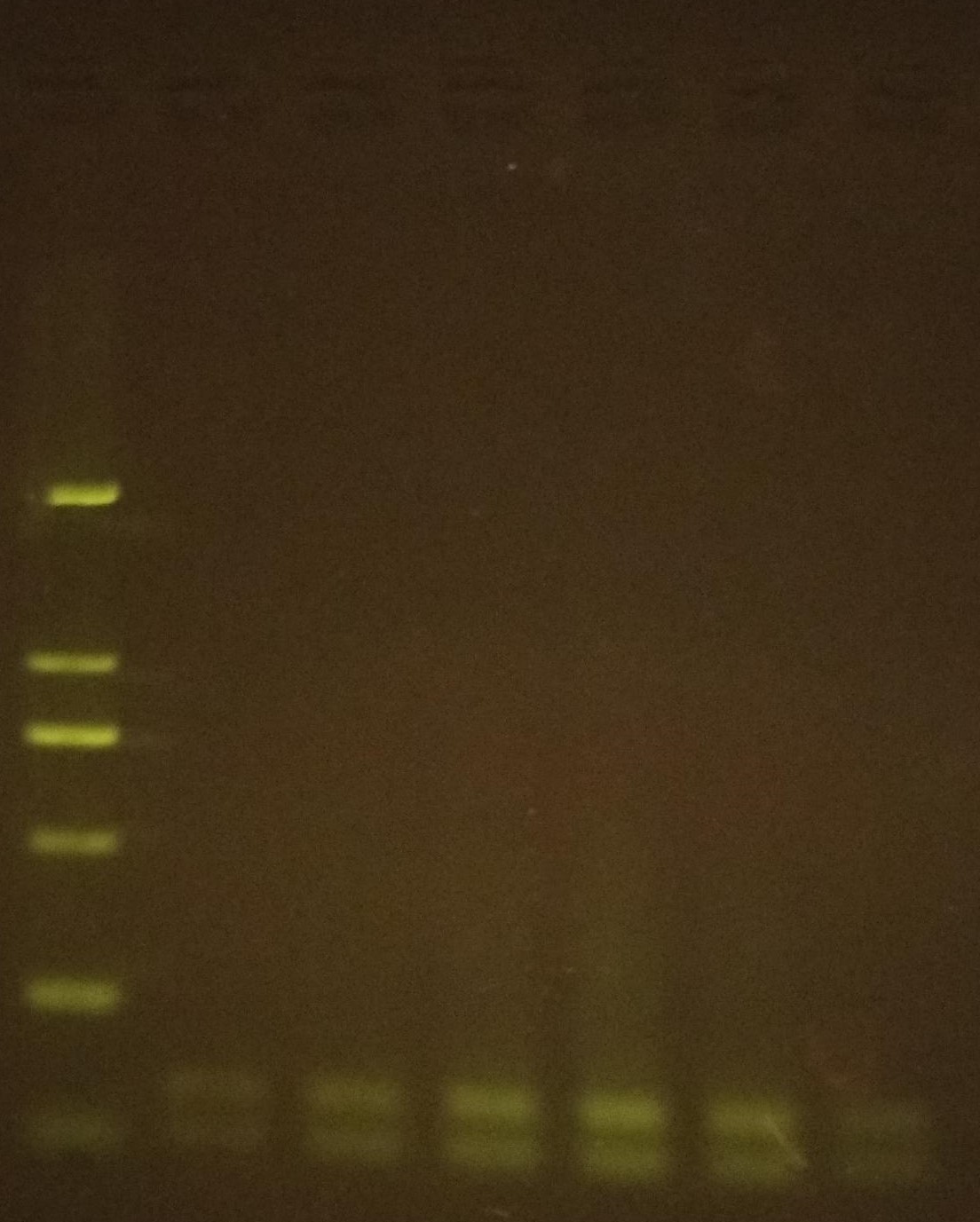

Supplement: S1 Raw images — (ZIP) [file pone.0251119.s001.zip › Figue 2-A.jpg]

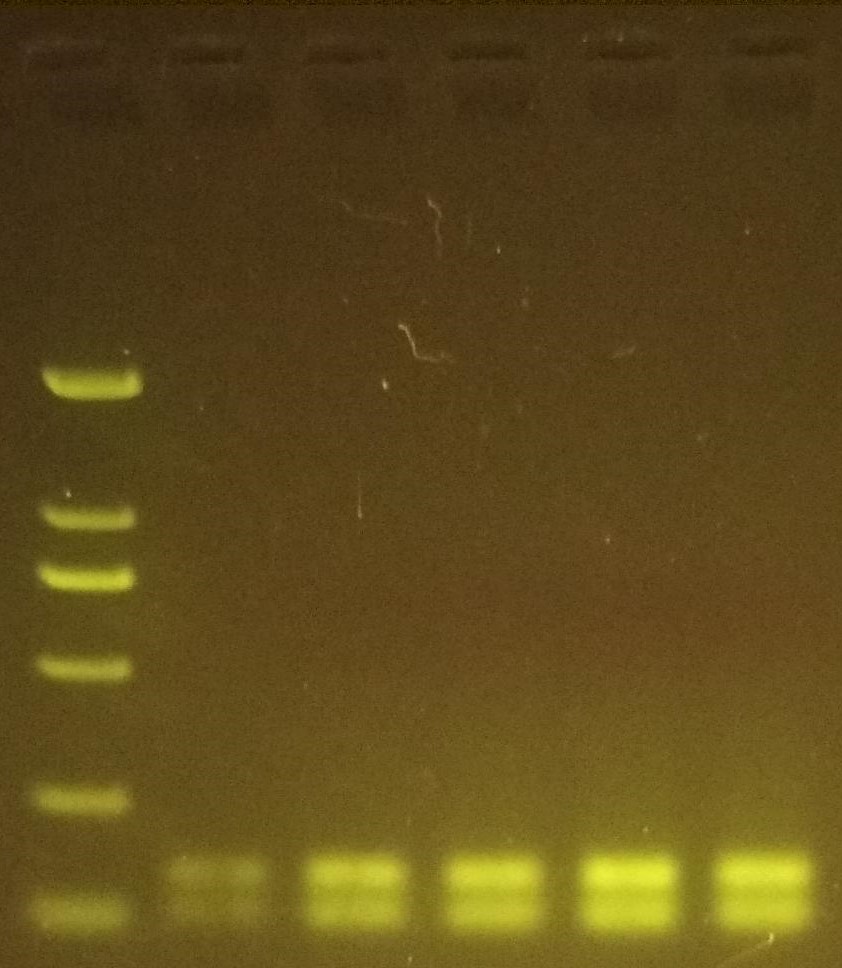

Supplement: S1 Raw images — (ZIP) [file pone.0251119.s001.zip › Figue 2-B.jpg]

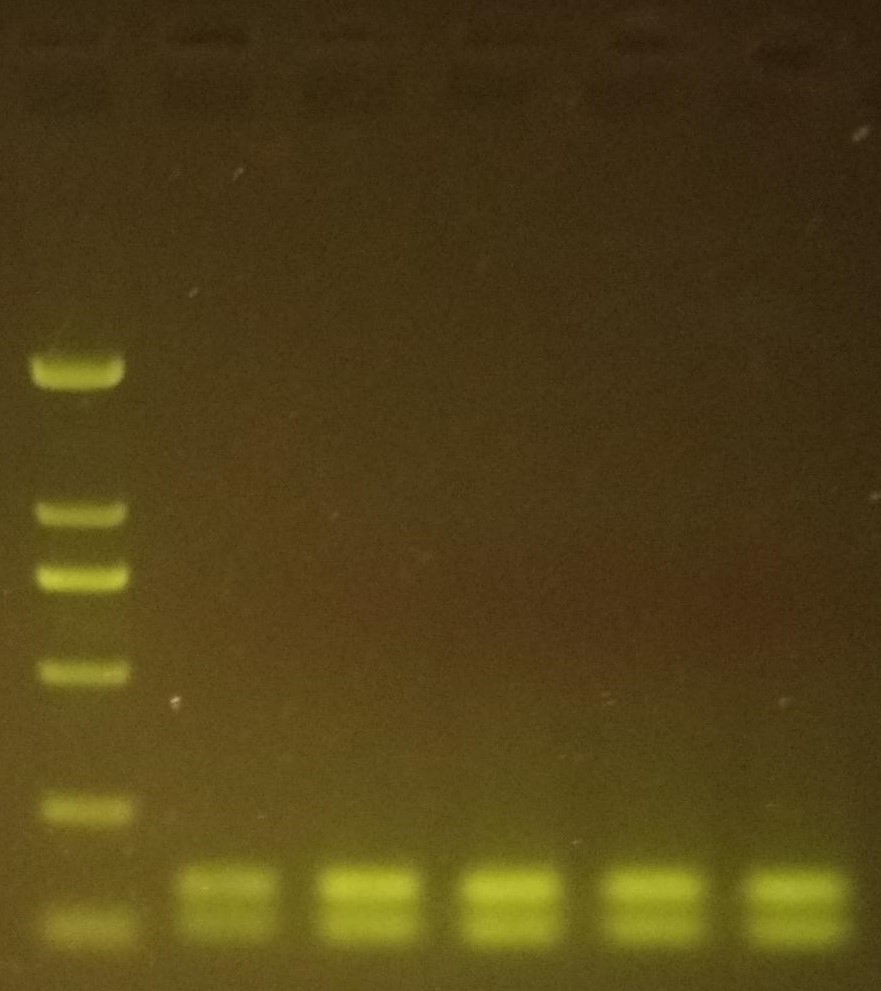

Supplement: S1 Raw images — (ZIP) [file pone.0251119.s001.zip › Figue 2-C.jpg]

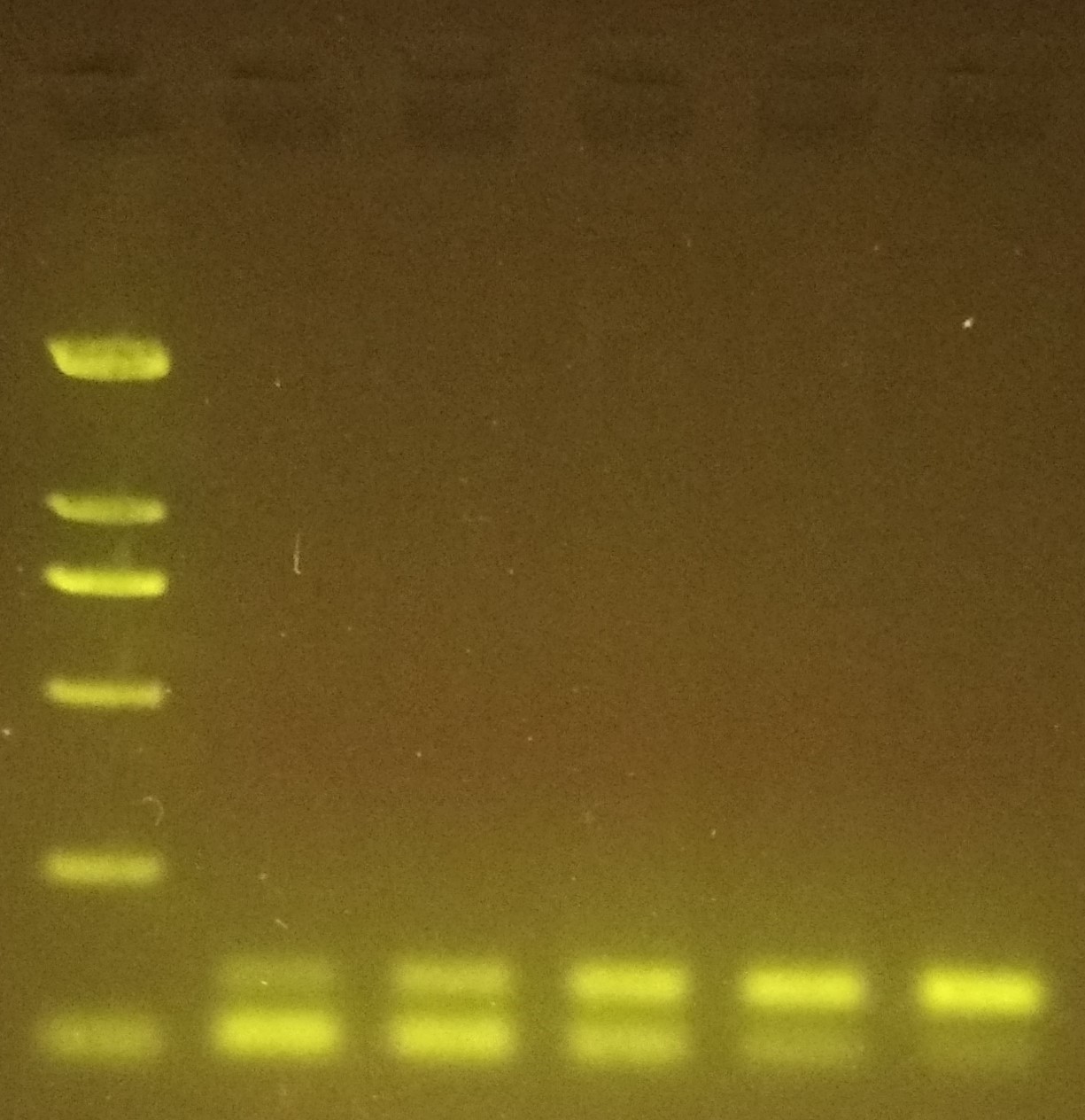

Supplement: S1 Raw images — (ZIP) [file pone.0251119.s001.zip › Figue 2-D.jpg]

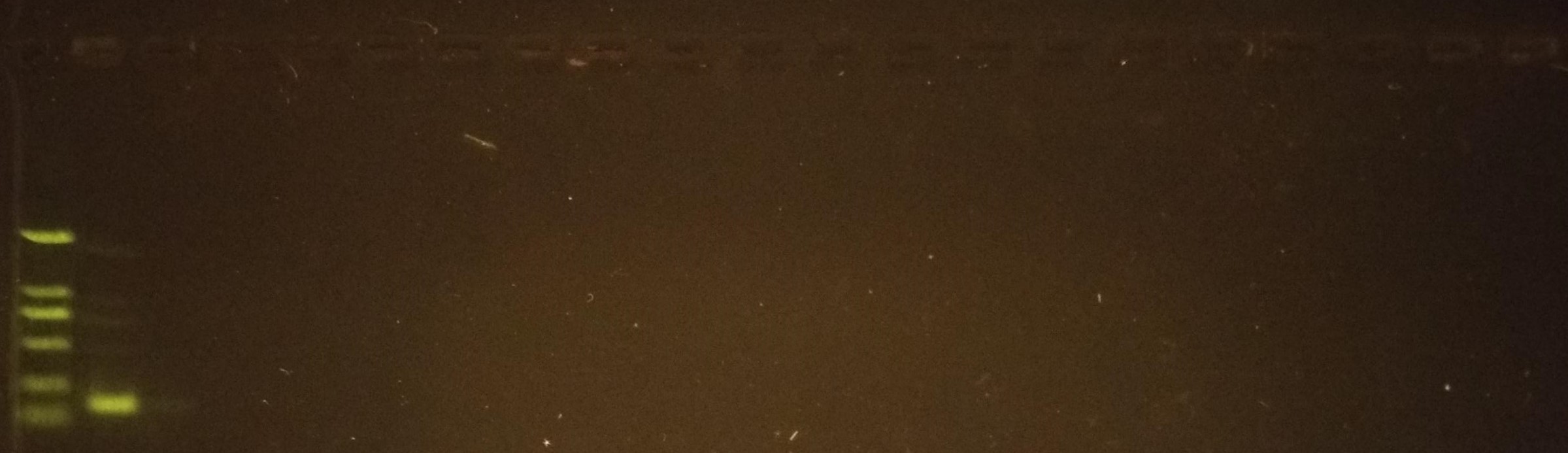

Supplement: S1 Raw images — (ZIP) [file pone.0251119.s001.zip › Figue 1-A.jpg]

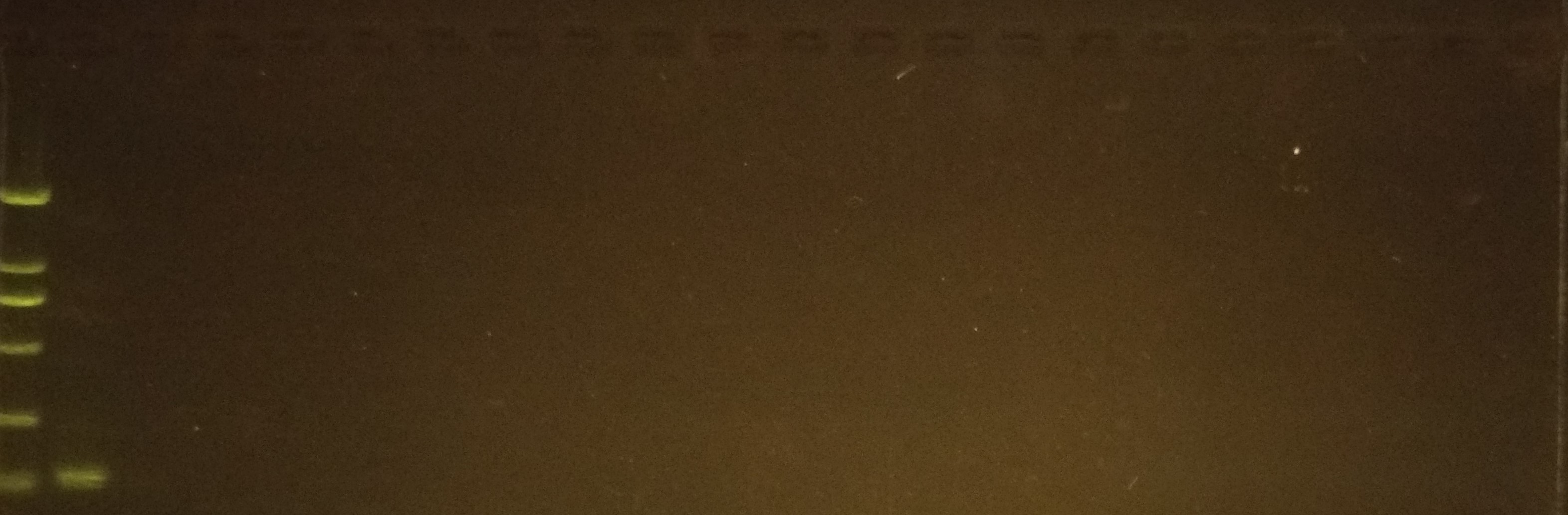

Supplement: S1 Raw images — (ZIP) [file pone.0251119.s001.zip › Figue 1-B.jpg]

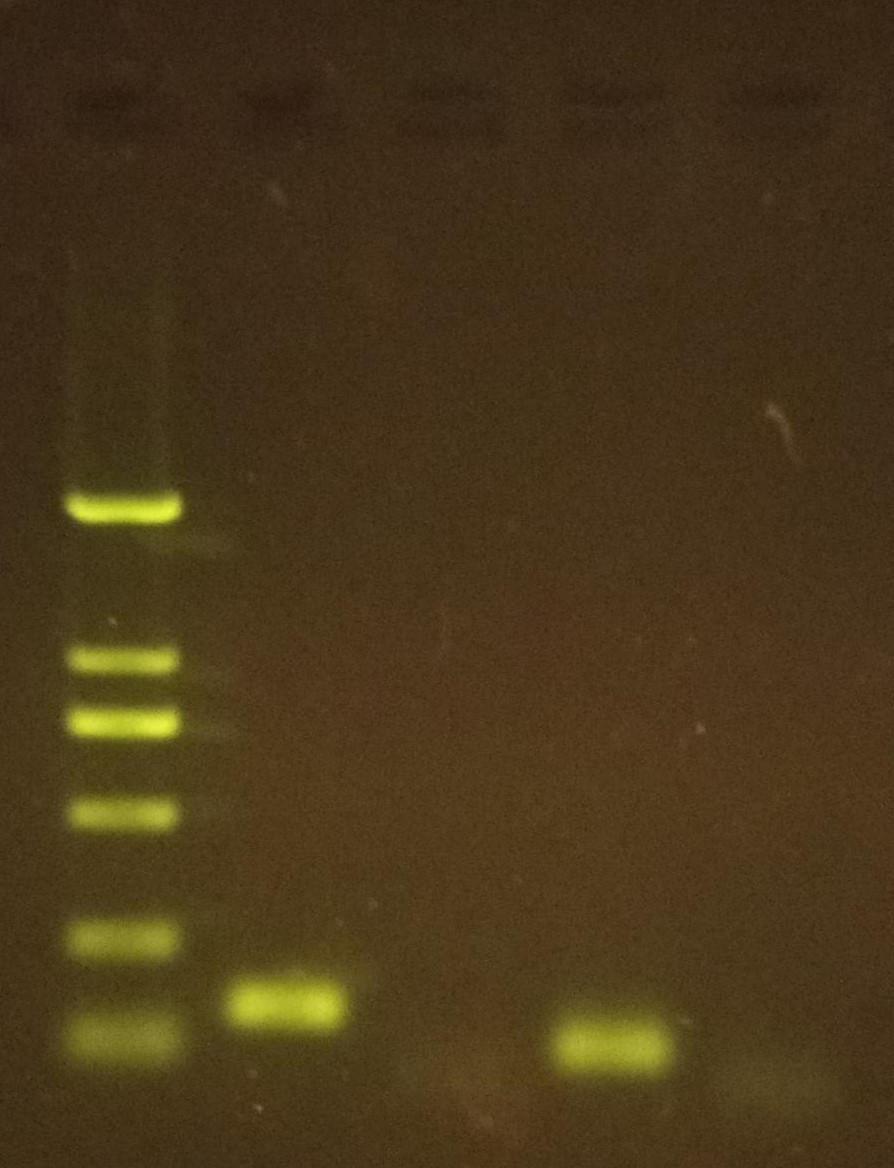

Supplement: S1 Raw images — (ZIP) [file pone.0251119.s001.zip › Figue 1-C.jpg]

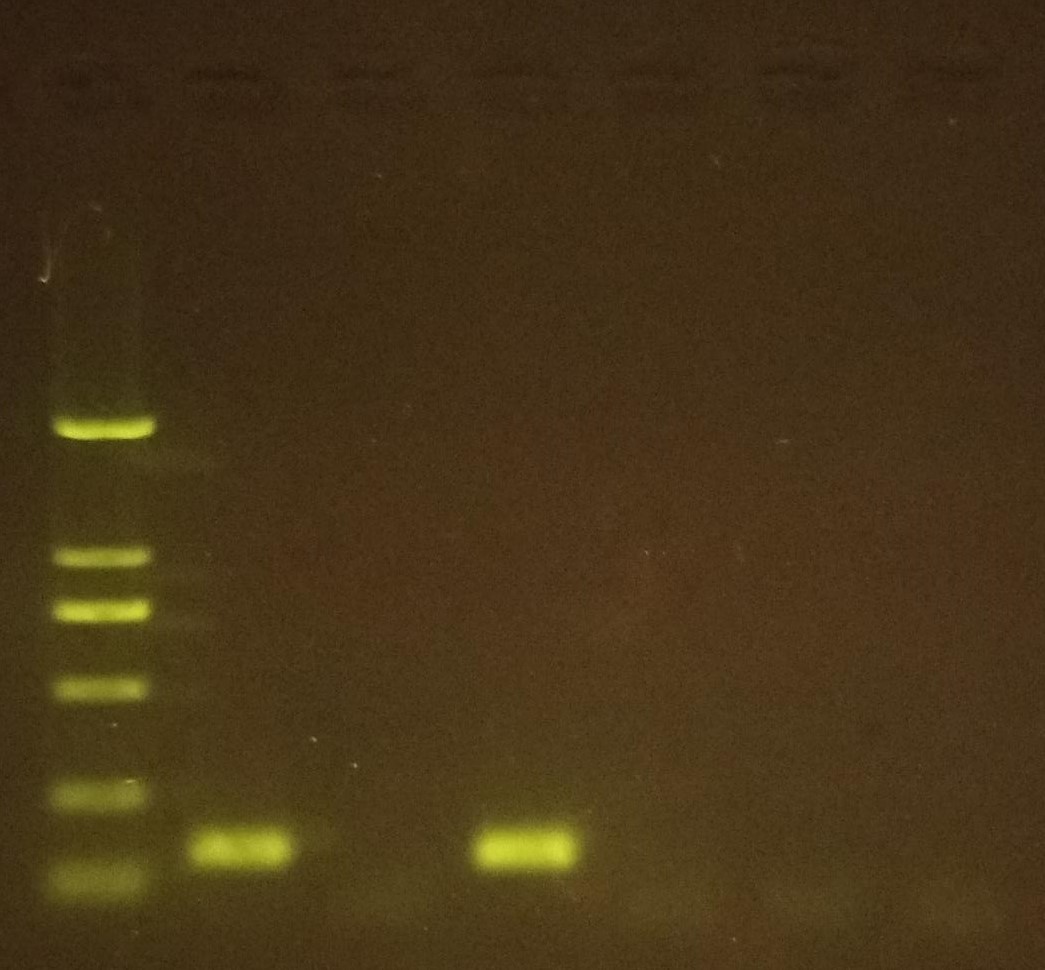

Supplement: S1 Raw images — (ZIP) [file pone.0251119.s001.zip › Figue 1-D-1.jpg]

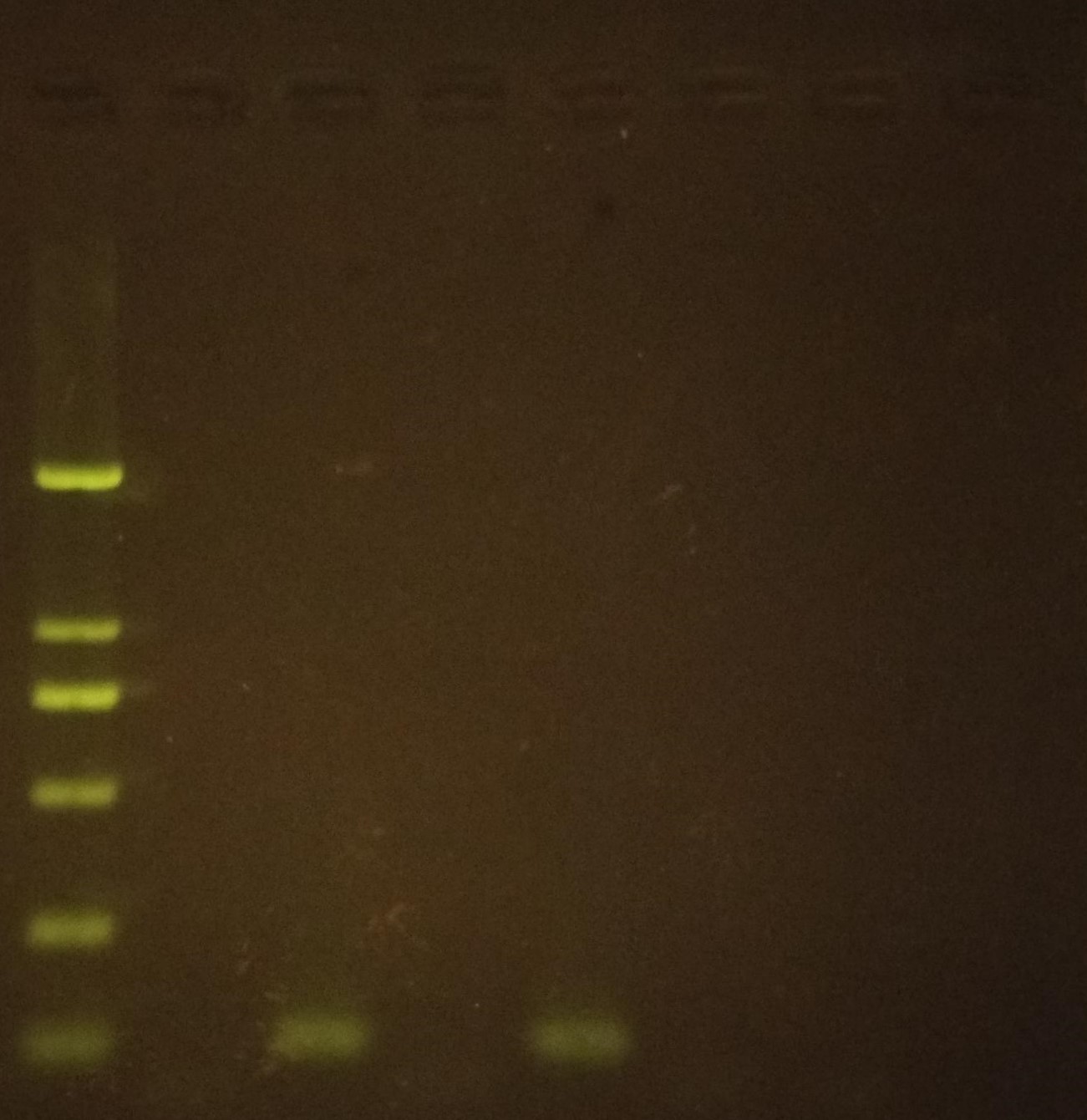

Supplement: S1 Raw images — (ZIP) [file pone.0251119.s001.zip › Figue 1-D-2.jpg]
